# Antigenic Evolution Characteristics and Immunological Evaluation of H9N2 Avian Influenza Viruses from 1994–2019 in China

**DOI:** 10.3390/v14040726

**Published:** 2022-03-30

**Authors:** Qingzheng Liu, Lingcai Zhao, Yanna Guo, Yongzhen Zhao, Yingfei Li, Na Chen, Yuanlu Lu, Mengqi Yu, Lulu Deng, Jihui Ping

**Affiliations:** MOE Joint International Research Laboratory of Animal Health and Food Safety, Engineering Laboratory of Animal Immunity of Jiangsu Province, College of Veterinary Medicine, Nanjing Agricultural University, Nanjing 210095, China; vetliuqingzheng@126.com (Q.L.); 2018207020@njau.edu.cn (L.Z.); gyn13655183568@163.com (Y.G.); 2019807145@stu.njau.edu.cn (Y.Z.); 2019107060@njau.edu.cn (Y.L.); 2020207021@njau.edu.en (N.C.); 15617938913@163.com (Y.L.); YMQ13515127867@163.com (M.Y.); dll980620@163.com (L.D.)

**Keywords:** avian influenza viruses, H9N2, hemagglutinin, antigenic evolution

## Abstract

The H9N2 subtype avian influenza viruses (AIVs) have been circulating in China for more than 20 years, attracting more and more attention due to the potential threat of them. At present, vaccination is a common prevention and control strategy in poultry farms, but as virus antigenicity evolves, the immune protection efficiency of vaccines has constantly been challenged. In this study, we downloaded the hemagglutinin (HA) protein sequences of the H9N2 subtype AIVs from 1994 to 2019 in China—with a total of 5138 sequences. The above sequences were analyzed in terms of time and space, and it was found that h9.4.2.5 was the most popular in various regions of China. Furthermore, the prevalence of H9N2 subtype AIVs in China around 2006 was different. The domestic epidemic branch was relatively diversified from 1994 to 2006. After 2006, the epidemic branch each year was h9.4.2.5. We compared the sequences around 2006 as a whole and screened out 15 different amino acid positions. Based on the HA protein of A/chicken/Guangxi/55/2005 (GX55), the abovementioned amino acid mutations were completed. According to the 12-plasmid reverse genetic system, the rescue of the mutant virus was completed using A/PuertoRico/8/1934 (H1N1) (PR8) as the backbone. The cross hemagglutination inhibition test showed that these mutant sites could transform the parental strain from the old to the new antigenic region. Animal experiments indicated that the mutant virus provided significant protection against the virus from the new antigenic region. This study revealed the antigenic evolution of H9N2 subtype AIVs in China. At the same time, it provided an experimental basis for the development of new vaccines.

## 1. Introduction

H9N2 influenza viruses were identified as a low-pathogenicity pathogen, but they have caused significant economic losses since they could reduce egg production and have been known to co-infect with other pathogens [1,2]. Turkey was one of the most susceptible species to the H9N2 virus, and its infection is usually accompanied by high morbidity and mortality [3]. In addition, reports showed that small poultry (chicken, duck, quail, partridge, pheasant, etc.) have a higher isolation rate of H9N2 subtype AIVs in the live poultry market [4,5]. In 1994, this subtype of the virus was first isolated from chickens in Guangdong province, China, and later found in other provinces [5,6,7]. H9N2 viruses can spread across the host, and, meanwhile, can promote infection and replication in human host cells due to the pH stability and receptor binding specificity of the HA protein [8,9,10,11]. In recent years, several other subtypes of influenza viruses (such as H7N9, H5N1, H10N8, and H5N6) were also related to the H9N2 viruses [12,13,14,15]. Therefore, several countries vaccinated poultry with conventional inactivated vaccines as a control measure [16,17]. However, H9N2 viruses consistently reduced vaccine protection efficiency due to antigenic drift [18].

Mutations in HA amino acid sites may cause changes in the physical characteristics of the epitope (shape, charge, polarity, etc.), thereby causing changes in antigenicity; this is a traditional immune escape mechanism of influenza viruses [19]. A hemagglutination inhibition assay (HI) is usually used to assess the antigenicity of influenza viruses [20,21]. Existing research has mainly identified the sites that affect antigenicity, which are near the receptor-binding region of HA. Mutations at these sites may lead to changes in epitope structure, glycosylation sites, and receptor affinity, thus affecting virus antigenicity [22,23].

China has implemented a long-term vaccination program in chicken farms since 1998 [16,24]. Vaccination (usually in the form of inactivated vaccines) has been shown to be effective in reducing clinical disease and production losses. Still, it has not been able to control the spread of the H9N2 virus completely. The main reason is the antigenic drift caused by poor vaccine antigenic match [25,26,27,28]. Matching vaccine strains with currently circulating strains is the key to successful immunization. Therefore, more research will be needed to develop alternative vaccination strategies and systems to control the spread of the H9N2 virus more effectively [29,30,31,32]. In the present study, we performed a temporal analysis of the HA1 protein of H9N2 strains in China from 1994 to 2019, and we found that it was different before and after 2006. Then we compared the HA1 protein sequences of the H9N2 virus around 2006 as a whole and screened out 12 different amino acid positions. The cross-hemagglutination inhibition test indicated that mutations at these sites can transform the parental strain from the old antigenic region to the new antigenic region. Animal experiments have found that the mutant virus had a specific immunoprotective effect on the virus, which comes from the new antigenic region. This study helps in providing an experimental basis for the development of new vaccines.

## 2. Materials and Methods

### 2.1. Virus

A/CK/JS/18325/18 (18325), A/CK/JS/19027/19 (19027), A/CK/JX/19166/19 (19,166), A/CK/WJ/57/18 (WJ57), A/CK/HN/98 (HN98), A/CK/SD/6/96 (SD96), and A/CK/SH/F/98(F98) were gifted by Jiangsu Lihua Animal Husbandry Co. Ltd. All viruses were propagated in 10-day-old specific-pathogen-free (SPF) embryonated chicken eggs. The allantoic fluid was harvested and stored at −80 °C until use.

### 2.2. Sequence Alignment and Phylogenetic Analysis

5138 completed HA amino acid sequences of H9N2 viruses were downloaded from the Influenza Research Database (IRD) from 1994 to 2019, which were updated to the latest available data in the database. Taking HA1 protein as the research object (removing the signal peptide sequence), the sequences were aligned using Bioedit software 7.1.3.0, and the alignment method was Clustal W Multiple alignments. The genetic analysis software MEGA 7.0.14 was used to draw a maximum likelihood phylogenetic tree with 1000 bootstrap replicates using RaxmlGUI 2.0 [33].

### 2.3. Phylogeographic Analyses

Based on the analysis above, we continued to conduct a more specific analysis of the downloaded sequences. First, we selected the sequences of the strains in each year from 1994 to 2019 and counted the number of sequences in each branch; secondly, we counted the number of sequences in each branch according to the different provinces. According to the analysis results, we conducted an overall alignment analysis of the sequences before and after 2007, and selected mutation sites based on the standard that the difference in the proportion of amino acids was more significant than 10%.

### 2.4. Structural Modeling of HA Proteins

To better characterize the amino acid variations in the HA protein of H9N2 viruses, we mapped the mutated sites to a predicted two-dimensional (2D) HA structure via homology modeling by using SWISS-MODLE and PyMOL software (http://pymol.sourceforge.net/, accessed on 1 May 2020), as reported previously [34]. Pymol software was used to draw the three-dimensional structure of the protein; different colors were used to indicate the receptor binding sites (RBS) and mutation sites on the surface of the HA protein.

### 2.5. Virus Rescue

The full-length HA gene of A/chicken/Guangxi/55/2005 (GX55) (accession number: EU086245) was synthesized by GenScript Biotech Corp (Wuhan, China). Then, the HA gene of GX55 was cloned into the RNA polymerase vector pHH21. Virus rescue was performed by the 12-plasmid reverse genetic system in the A/PuertoRico/8/1934 (PR8) backbone as previously described [35]. A total of two viruses were reused, which were GX55/HA wild-type reassortant virus (R-WT/PR8) and GX55/HA-mutant reassortant virus (R-Mut/PR8). R-Mut/PR8 was based on the 6 internal genes backbone of PR8 and the surface glycoproteins from GX55 with 12 amino acids mutations in the HA gene.

### 2.6. HI Assay and Antigenic Cartography

The HI assay was used to antigenically characterize the H9N2 viruses. Antisera of 9 selected H9N2 viruses (R-WT/PR8, R-Mut/PR8, A/CK/JS/18325/18, A/CK/JS/19027/19, A/CK/JX/19166/19, A/CK/WJ/57/18, A/CK/HN/98 A/CK/SD/6/96, A/CK/SH/F/98) were generated in 3-week-old SPF chickens. Each chicken was vaccinated with 0.4 mL Freund’s-adjuvanted, inactivated whole virus vaccines. Sera from vaccinated chickens were collected 21 days after the vaccination. The HI test was performed as previously described [36]. According to the abovementioned cross hemagglutination inhibition test results, antigenic cartography (http://www.antigenic-cartography.org/ accessed on 18 March 2022) was used to mathematically transform the HI titer to form a map of antigenic distance.

### 2.7. Immune Protection Experiment

To further study the protective immunity induced by R-Mut/PR8 vaccines against the GX55 and A/CK/JX/19166/19 strains, which represent the old antigen group and the new antigen group, respectively (*n* = 20/group), 3-week-old SPF chickens were immunized with inactivated vaccines (0.4 mL/chicken). Three weeks post-vaccination, 1 mL of blood was collected from each chicken to make anti-sera. The antibody titer was evaluated by an HI assay in order to monitor the antibody levels of the vaccinated chickens before the challenging study. Each chicken in the different groups was intranasally injected with 10^6^ TCID_50_ of the H9N2 virus. Trachea swabs from each infected chicken were collected on days 3, 5, 7, and 9 post-challenge. For the virus shedding test, a total of 0.1 mL of each swab sample supernatant was inoculated into the allantoic cavity of 10-day-old chicken embryos. The samples collected in the 3 days post infection (dpi) were titrated by the 50% infectious egg dose (EID_50_) using the Reed and Muench method [37].

The chickens were handled humanely according to the rules described by the Animal Ethics Procedures and Guidelines of the People’s Republic of China and the Institutional Animal Care and Use Committee of Nanjing Agricultural University.

### 2.8. Statistical Analysis

The student’s *t*-test was used to analyze the statistical comparison between the two groups. (ns = not significant, * *p* < 0.05, *** *p* < 0.001, **** *p* < 0.0001). Error bars represent standard error (±SEM). Statistical analysis was performed using GraphPad Prism version 8.0.

## 3. Results

### 3.1. Genetic Evolution of Domestic H9N2 HA1 Protein from 1994 to 2019

**Figure 1:**
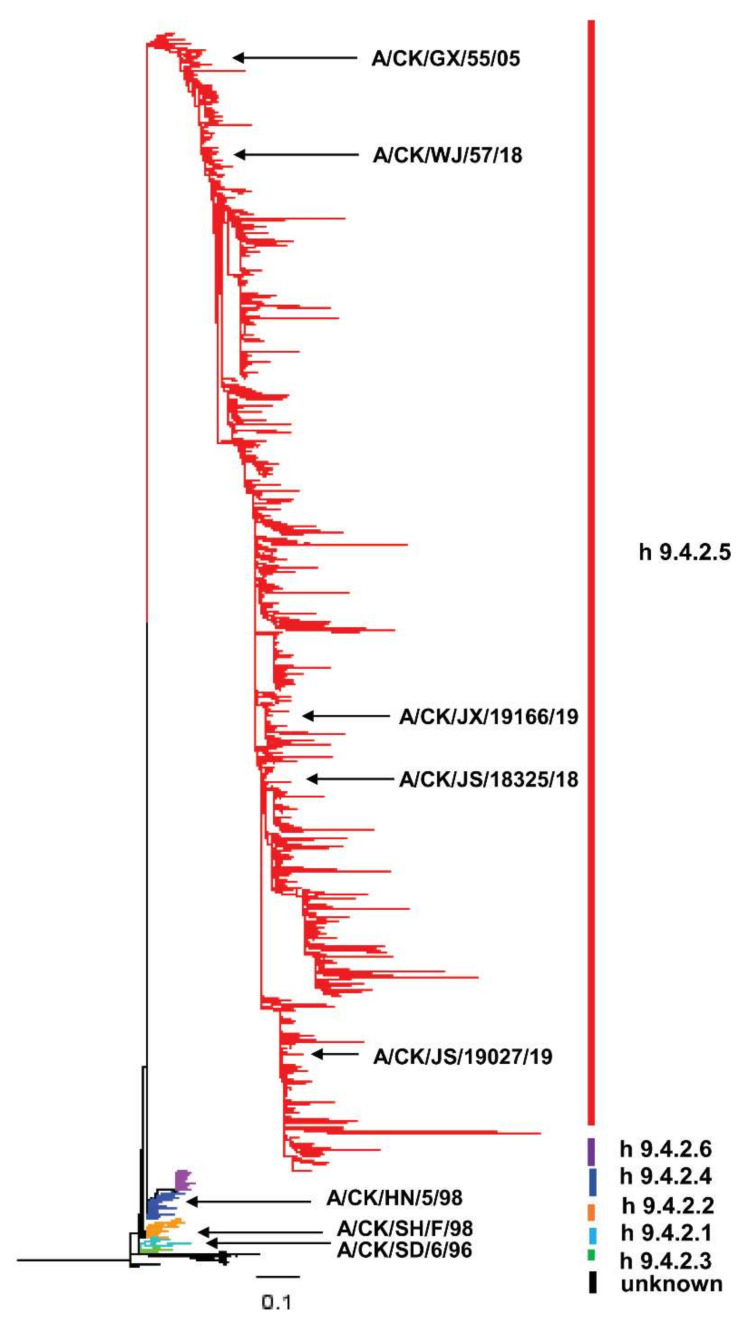
Phylogenetic evolutionary of HA protein of H9N2 AIV from 1994 to 2019 in China. Different epidemic branches were represented by different colors. CK: Chicken, GX: Guangxi, JX: Jiaxing, JS: Jiangsu, HN: Henan, SD: Shandong, WJ: Wujin, SH: Shanghai.

A total of 5138 HA full-length amino sequences of domestic H9N2 subtype strains from 1994 to 2019 in the Influenza Research Database (IRD) were downloaded and the HA1 protein sequences were intercepted. The phylogenetic tree is presented in Figure 1. According to the results of the genetic evolutionary analysis, it was found that the domestic H9N2 subtype AIVs have been mainly divided into 6 epidemic branches (h9.4.2.1, h9.4.2.2, h9.4.2.3, h9.4.2.4, h9.4.2.5, and h9.4.2.6) since 1994. Meanwhile, we could see that the domestic epidemic branch with the largest proportion was h9.4.2.5, which represented the virus strain A/Chicken/Guangxi/55/2005 (GX55). The result exhibited the domestic prevalence of H9N2 subtype AIVs from 1994 to 2019 in China, and it indicated that GX55 could be used as a template for subsequent research.

### 3.2. The Temporal and Spatial Distribution of Genetic Evolution of HA1 Protein

**Figure 2:**
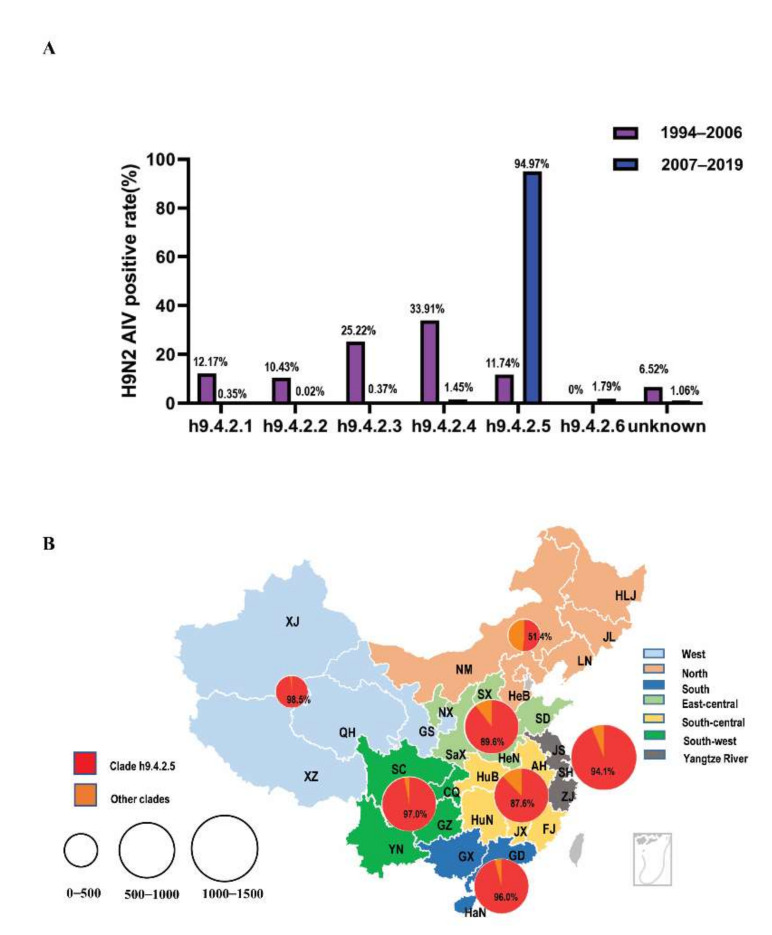
H9N2 AIV sampling sites and isolation rates in Chinese provinces from 1994–2019. (**A**) 23 provinces, 5 municipalities, and 4 minority autonomous regions in China were divided into 7 different regions: West (Gansu, GS; Xinjiang, XJ; Qinghai, QH; and Xizang, XZ; light blue). North (Heilongjiang, HLJ; Inner Mongolia, NM; Hebei, HeB; Liaoning, LN; and Jilin, JL; pink), South (Guangxi, GX; Guangdong, GD and Hainan, HaN; blue), East-Central (Shandong, SD; Shaanxi, SaX; Shanxi, SX; Henan, HeN and Ningxia, NX; light green), South-Central (Anhui, AH; Hubei, HuB; Jiangxi, JX; Hunan, HuN and Fujian, FJ; yellow), South-West (Sichuan, SC; Guizhou, GZ; Chongqing, CQ and Yunnan, YN; green), Yangtze River Delta (Jiangsu, JS; Shanghai, SH; and Zhejiang, ZJ; black). The red portion in each pie chart indicates the isolation rate of clade h9.4.2.5 in this region; the isolation rate of the other clades was represented by dark yellow. Different sample sizes were represented by circles of different sizes. (**B**) The AIV positive rates from 1994–2006 were represented by purple; the AIV positive rates from 2007–2019 were represented by blue. The regions included West, South, North, East-Central, South-Central, South-West, and Yangtze River. The numbers on the column represent the AIV isolation rates.

Based on the above results, we continued to conduct a more specific analysis of the downloaded sequence. First, we selected the strain sequences of each year from 1994 to 2019 and then counted the sequences number of each branch (Figure 2A). Secondly, we counted the sequences number of each branch according to the different provinces in China (Figure 2B); the detailed data is shown in Tables S1 and S2. In 2006 and before, the domestic epidemic branches were relatively diversified. Although h9.4.2.4 was the dominant branch in 1999–2002 and 2004, its advantage was not obvious. After 2006, the domestic epidemic branch was dominated by h9.4.2.5 and the epidemic advantage of this branch was obvious. The h9.4.2.6 branch appeared in 2009, but it didn’t form a dominant popular branch. From Table S2 we can see that, except for Gansu Province and Inner Mongolia Province, the epidemic branches in other provinces were all h9.4.2.5.

### 3.3. Screening of 12 Different Mutant Amino Acid Sites from the Sequences around 2006

The sequences were divided into two groups around 2006 and overall comparison and analysis of the two sets of sequences were performed. The screening was performed based on the standard that the difference in the proportion of each amino acid site, and a total of 12 different amino acid sites (positions 72, 109, 135, 146, 149, 162, 182, 202, 216, 217, 236, and 238, H9 numbering) were screened. The detailed results are shown in Table S3. Then, we analyzed the distribution of the mutant combination in the natural. After this, we found that these 12 amino-acid-mutated viruses exist in the natural environment.

Reassortants with internal genes from the PR8 virus but the NA and mutant HA gene from the corresponding wild-type H9N2 virus (GX55) were generated (R-Mut/PR8). Next, we used Pymol software to display these mutation sites (Figure S1). The H9N2 subtype HA protein has a pocket-like region called the receptor binding site (RBS), which is located at positions 92, 128, 132, 143, 173, 184, 185, 214, 215, 218, and 219 (H9 numbering) and marked with a yellow color in Figure S1.

### 3.4. Antigenicity Analysis of the R-Mut/PR8 and Other Viruses

**Figure 3:**
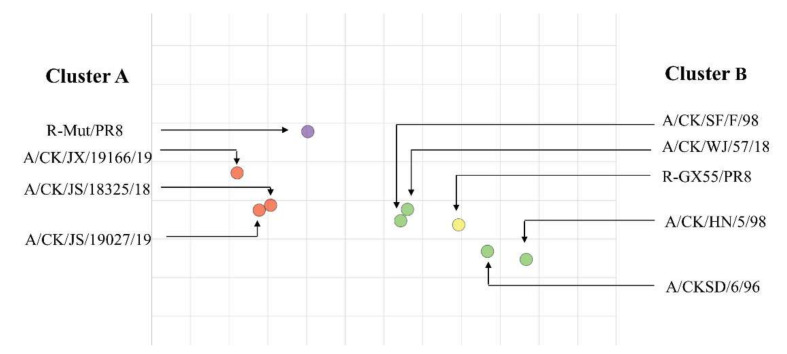
Antigen map of recombinant virus and wild-type strains. The solid circles indicated the positions of the virus. Clusters A and B were represented by red and green solid circles, respectively. R-Mut/PR8 and R-GX55/PR8 were represented by purple and yellow solid circles The spacing of the grid lines corresponds to the HI unit, which was equivalent to twice the difference in the HI measurement. CK: chicken, JX: Jiaxing, JS: Jiangsu, HN: Henan, SD: Shandong, WJ: Wujin, SH: Shanghai.

The antisera of two recombinant viruses (R-WT/PR8, R-Mut/PR8) and seven wild isolated viruses (A/CK/JS/18325/18, A/CK/JS/19027/19, A/CK/JX/19166/19, A/CK/HN/5/98, A/CKSD/6/96, A/CK/WJ/57/18, A/CK/SH/F/98) were generated according to the method described previously. These viruses and antisera were subjected to cross-hemagglutination inhibition experiments. These data were quantitatively analyzed using Antigenic Cartography software and drawn into an antigenic map (Figure 3).

It can be found from Table S4 that the HI titer of each antiserum and its corresponding virus was relatively high, which was equal to or greater than 2^8^. The HI titer of R-WT/PR8 reacted with the antiserum of 18325, 19027, and 19166, which was only 2^3^ and showed that the GX55 strain had a poor ability to cross-react with the wild strains that have been popular in recent years. Different from R-WT/PR8, the HI titer of R-Mut/PR8 reacted with the antiserum of 18325, 19027, and 19166, which was higher—ranging from 2^7^ to 2^9^. However, the HI titer of R-WT/PR8 reacting with R-Mut/PR8 is relatively low, only 2^5^. These results indicated that the cross-reaction of a mutant recombinant virus became weaker when reacting with its parent virus (GX55), but it had a stronger cross-reaction with the wild strain isolated in recent years.

From Figure 3, the viruses participated in the test can be intuitively divided into two antigen groups (marked with red and green colors). The antigenic region of R-WT/PR8 was close to F98, HN98, SD96, and WJ57. However, R-Mut/PR8 was closed to the antigenic region of 18325, 19027, and 19166. In conclusion, firstly, GX55 and the currently popular H9N2 wild strains formed two antigenic groups, and, secondly, the mutation combination of R-Wut/PR8 changed the distribution of the antigenic region of the parent virus, resulting in the development of antigen groups that moved toward the new antigenic group.

### 3.5. R-Mut/PR8 Inactivated Vaccine Provides Adequate Immune Protection against 19166 Strain

The R-Mut/PR8 recombinant virus was combined with the above experimental results to prepare an inactivated vaccine. To study its immunoprotective effects on new and old strains, we selected R-WT/PR8 and 19166 inactivated vaccines as the positive control group, with the PBS group as the negative control group. The immunoprotection test was carried out according to the method described previously.

From Table 1, we can see that in the PBS control group the virus shedding of chickens challenged by 19166 can be extended to day 7, but the virus isolation rate of chickens challenged by 19166 on day 7 was only 10%. In this group, the virus shedding of chickens challenged by GX55 lasted until day 5. The chickens challenged by these 2 viruses in this group had a virus isolation rate of 100% on day 3 and day 5. In the R-WT/PR8 vaccine group, the virus isolation rate of chickens infected with GX55 was only 20% on day 5, and no shed virus was isolated on day 7 and day 9. This result indicated that the GX55 inactivated vaccine is effective against the parental virus. However, in this group, the shed virus isolation rate challenged by 19166 on day 5 was 60%, which was much higher than the 19166 vaccination group. This result indicated that the protective effection of the R-WT/PR8 vaccine against 19166 was not as effective as its parental vaccine. Similarly, the 19166 vaccination group showed adequate immune protection against the parental virus (19166), but the immune protection effectiveness against GX55 was weaker than that. It can be found that the shed virus isolation rate of the R-Mut/PR8 vaccine group challenged by GX55 on day 5 was 40%, which was the same as the result of the 19166 vaccination group. In the R-Mut/PR8 vaccine group, the virus isolation rate on day 5 challenged by 19166 was 40%, which was lower than 60% of the R-WT/PR8 group, indicating that the R-Mut/PR8 virus had a strong immune protective effect against the 19166 strain.

**Figure 4:**
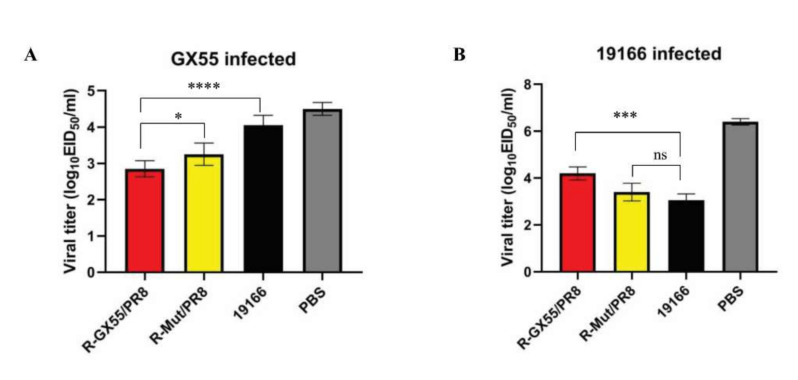
Protective efficacy of R-Mut/PR8 virus. 3-week-old SPF chickens were immunized with inactivated vaccines (0.4 mL/chicken). Three weeks post-vaccination, each chicken in different groups was intranasally injected with 10^6^ TCID_50_ of the H9N2 virus. The samples collected at 3 days post infection (dpi) were titrated by the 50% infectious egg dose (EID_50_). (**A**): The immune protective efficacy of candidate vaccines against GX55 strain. (**B**): The immune protective efficacy of candidate vaccines against 19166 strain. Statistical differences were labeled according to t-test in GraphPad Prism 8 (ns = not significant, * *p* < 0.05, *** *p* < 0.001, **** *p* < 0.0001).

It can be seen from Figure 4 that the virus titer of the PBS control group challenged by GX55 and 19166 was higher than that of the other three groups. These results indicated that all virus replication was inhibited to a certain extent. In the 19166 vaccination group, three days after GX55 was infected, the virus titer of the trachea swabs was significantly higher than that of the R-WT/PR8 vaccination group. The virus titer of the samples from the R-Mut/PR8 vaccination group was also significantly higher than that of the R-WT/PR8 vaccination group, while the difference multiple is smaller compared with the 19166 vaccination group. 

In summary, challenged by the GX55 virus, R-Mut/PR8 inactivated vaccine showed a weaker protective effect than the R-WT/PR8 positive control group, but it was more significantly effective than the 19166 vaccines. As well, R-Mut/PR8 inactivated vaccine had adequate immune protection against the 19166 strain. This result showed that the combination of R-Mut/PR8 mutations would enhance the immune protective effect of the recombinant virus against the 19166 strain, which represented a new antigenic group. 

## 4. Discussion

The H9N2 subtype AIVs are prevalent in most parts of Asia, the Middle East, and North Africa. They cause serious economic losses to the poultry industry through high transmission and morbidity, and they can damage human health through mutation and recombination [38]. Therefore, H9N2 viruses are considered to be a potential threat for a pandemic. Poultry vaccination is a key national measure for epidemic disease control, but the effectiveness of vaccines has always been challenged by the antigenic variant of the viruses [19,39].

In recent years, most of the H9N2 viruses isolated in China belonged to the h9.4.1 and h9.4.2 lineages. Previously, these two lineages were named as G1-like and Y280/G9-like clades. During the past few years, the h9.4.2 lineage (Y280/G9) has been dominant in China; however, this lineage has recently evolved into two major lineages, h9.42.5 and h9.4.2.6 [27]. Currently, h9.4.2.5 is the dominant lineage in China [40]. In this study, the HA1 amino acid sequences of the H9N2 viruses were taken as the research object, then its prevalence from 1994 to 2019 in China was analyzed. According to the analysis results, the sequences around 2006 were divided into two groups and 12 different amino acid positions between two groups were screened out. Using the HA protein of the representative strain (GX55) in the current main epidemic branch h9.4.2.5 as a template, the mutant plasmid was successfully constructed, and the mutant virus was rescued. The antigenic changes of the mutant virus were studied through the cross hemagglutination inhibition assay and animal experiments verified the immune protective effect of the mutant virus. Previous researchers have reported some of the amino acid sites screened in this study. For example, Peacock et al. have shown that mutations of G72E, D135G, Q146H, G149D/K, L150S, T182R, N183T, and I217L/MQ have amino acid residues that can cause antigen escape of H9N2 AIVs [38,40,41,42]. 

There have been many studies on the amino acid mutations of the H9N2 AIV HA protein in the early stages, and many amino acid mutation sites have been discovered that can change the antigenicity of H9N2 AIVs. However, in the natural environment, its HA protein always exists in the form of a combination of multiple site mutations, and thus it is also of great significance to study the effect of multi-site combination mutations on virus antigenicity. GX55 was used as a template to complete the mutation of the combination in different amino acid positions. With PR8 internal genes as the backbone, the mutant virus was rescued using a 12-plasmid reverse genetic system. The rescued recombinant virus was subjected to a cross hemagglutination inhibition reaction with the H9N2 wild strain that was isolated in recent years, and the antigen map was drawn. From this result, it can be found that although the current domestic H9N2 strain is in the same branch with GX55 (h9.4.2.5), there is an apparent antigenic difference between GX55 and the newly isolated strains; perhaps it was due to the natural evolutionary trend of the H9N2 viruses or the adaptive mutation under the immunization pressure of different vaccine strains, which led to the change of the dominant lineage in China around 2006. However, the recombinant mutant R-Mut/PR8 was relatively close to the antigenic region of the newly isolated strains, such as 19166. Based on this result, a new conjecture suggests that R-Mut/PR8 virus can be prepared as an inactivated vaccine against newly isolated strains.

At present, the prevention and control of H9N2 AIVs in poultry mainly depends on the injection of inactivated vaccines. However, due to the weak matching ability between vaccines and antigens caused by antigenic drift, the H9N2 AIVs have been difficult to prevent and control in recent years [28]. According to the previous research of our laboratory, four strains of H9N2 virus (19027, 19166, 18325, WJ57) were isolated in Jiangsu Province and Zhejiang Province. Combined with the results of previous experiments, the R-Mut/PR8 recombinant virus we selected to prepare an inactivated vaccine. Since 19166 was the latest strain isolated in our laboratory, its residues at the 12 amino acid positions were consistent with the dominant sequences after 2006 (Table S3), wherein we selected 19166 for animal experiments. Based on the monitoring results of virus shedding, it was found that the R-Mut/PR8 vaccine can effectively shorten the virus shedding time of the GX55 strain and the 19166 strain. The virus titer of the trachea swabs was tested on 3 dpi, and the R-Mut/PR8 vaccine can effectively reduce the ability of 19166 to replicate in chickens. This result indicated that the R-Mut/PR8 mutant virus greatly influenced the change in virus antigenicity, and the inactivated vaccine prepared from the R-Mut/PR8 mutant virus can effectively protect chickens from the 19166 strain, which was a new epidemic virus. Although this study can provide some theoretical support, these strains involved in this study cannot fully represent the epidemic characteristics of all viruses in the h9.4.2.5 lineage. Thus, further research is needed to explain this issue.

## Figures and Tables

**Table 1:** Virus shedding of trachea swabs in vaccinated chickens challenged by H9N2 virus.

Vaccines	HI Titers(log2)	Challenge Virus	Positive Viral Isolation Chickens/Total Chickens			
			Day 3	Day 5	Day 7	Day 9
R-WT/PR8	10.67 ± 0.82	GX/55 19166	10/10 10/10	2/10 6/10	0/10 0/10	0/10 0/10
19166	11.25 ± 0.66	GX/55 19166	10/10 10/10	4/10 2/10	0/10 0/10	0/10 0/10
R-Mut/PR8	11.4 ± 0.66	GX/55 19166	10/10 10/10	4/10 4/10	0/10 0/10	0/10 0/10
PBS	0	GX/55 19166	10/10 10/10	10/10 10/10	0/10 1/10	0/10 0/10

## Data Availability

Not applicable.

## Supplementary Materials

The following supporting information can be downloaded at: https://www.mdpi.com/article/10.3390/v14040726/s1, Figure S1: Position of the selected amino acids in HA 3D trimer structure, Table S1: The branches of H9N2 HA proteins each year in China from 1994 to 2019, Table S2: The space dynamics of the H9N2 HA protein in China from 1994 to 2019, Table S3: The different amino acids of HA sequences around 2006, Table S4: Results of cross hemagglutination inhibition test.
