# Supplementary material for: Antigenic Evolution Characteristics and Immunological Evaluation of H9N2 Avian Influenza Viruses from 1994–2019 in China
Source: Viruses. 2022 Mar 30;14(4):726. doi: 10.3390/v14040726 (PMC9031003; doi:10.3390/v14040726)
Supplement: Supplementary file 1 [file viruses-14-00726-s001.zip › viruses-1620641-supplementary.pdf]

viruses

MDPI

# Supplementary Materials

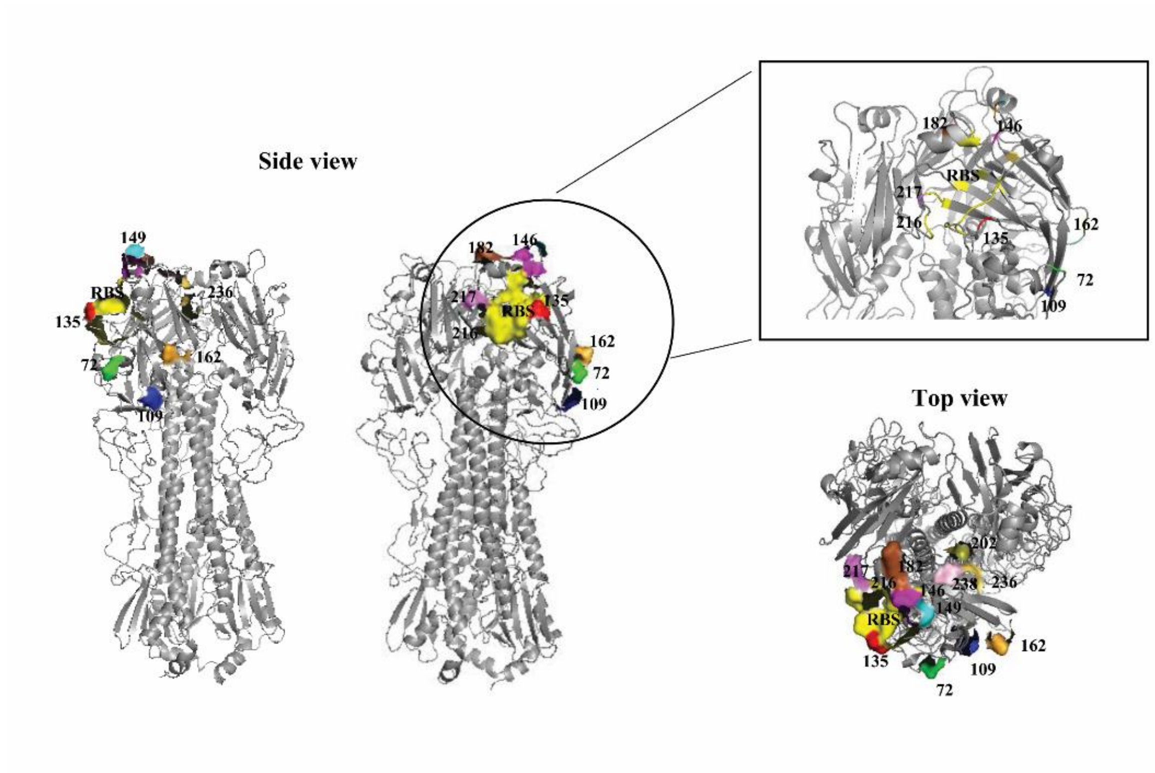


**Figure S1.** Position of the selected amino acids in HA 3D trimer structure. The 3D structure of the HA protein trimer was drawn with PyMOL. The receptor binding site was yellow. Position 72 was marked as green, position 109 was blue, position 135 was red, position 146 was purple, position 149 was cyan, position 162 was deep brown, position 182 was brown, position 202 was deep olive, position 216 was deep blue, position 217 was violet, position 236 was yellow orange, position 238 was light pink.

**Table S1.** The branches of H9N2 HA proteins each year in China from 1994 to 2019.

| Year | Total Number | Clade | | | | | | |
|---|---|---|---|---|---|---|---|---|
| | | h9.4.2.1 | h9.4.2.2 | h9.4.2.3 | h9.4.2.4 | h9.4.2.5 | h9.4.2.6 | Unknown |
| 1994 | 2 | 0 | 2 | 0 | 0 | 0 | 0 | 0 |
| 1995 | 0 | 0 | 0 | 0 | 0 | 0 | 0 | 0 |
| 1996 | 6 | 2 | 4 | 0 | 0 | 0 | 0 | 0 |
| 1997 | 7 | 1 | 3 | 2 | 0 | 0 | 0 | 1 |
| 1998 | 16 | 5 | 1 | 1 | 4 | 0 | 0 | 4 |
| 1999 | 22 | 5 | 2 | 7 | 8 | 0 | 0 | 0 |
| 2000 | 32 | 5 | 0 | 6 | 17 | 2 | 0 | 2 |
| 2001 | 40 | 3 | 5 | 14 | 14 | 3 | 0 | 1 |
| 2002 | 23 | 5 | 1 | 3 | 10 | 4 | 0 | 0 |
| 2003 | 19 | 2 | 4 | 3 | 4 | 5 | 0 | 1 |
| 2004 | 12 | 0 | 2 | 1 | 7 | 0 | 0 | 2 |
| 2005 | 27 | 0 | 0 | 17 | 4 | 3 | 0 | 3 |
| 2006 | 25 | 0 | 0 | 4 | 10 | 10 | 0 | 1 |
| 2007 | 107 | 1 | 1 | 6 | 36 | 57 | 0 | 6 |
| 2008 | 57 | 4 | 0 | 2 | 23 | 26 | 0 | 2 |
| 2009 | 94 | 0 | 0 | 1 | 5 | 85 | 1 | 2 |

| | | | | | | | | |
|---|---|---|---|---|---|---|---|---|
| 2010 | 154 | 1 | 0 | 2 | 3 | 136 | 10 | 2 |
| 2011 | 224 | 1 | 0 | 5 | 2 | 149 | 59 | 8 |
| 2012 | 156 | 2 | 0 | 1 | 0 | 125 | 11 | 17 |
| 2013 | 456 | 5 | 0 | 1 | 1 | 439 | 4 | 7 |
| 2014 | 204 | 0 | 0 | 0 | 0 | 196 | 1 | 7 |
| 2015 | 548 | 0 | 0 | 0 | 0 | 547 | 1 | 0 |
| 2016 | 738 | 1 | 0 | 0 | 0 | 736 | 1 | 0 |
| 2017 | 742 | 2 | 0 | 0 | 1 | 738 | 0 | 1 |
| 2018 | 1402 | 0 | 0 | 0 | 0 | 1402 | 0 | 0 |
| 2019 | 25 | 0 | 0 | 0 | 0 | 25 | 0 | 0 |
| Sum | 5138 | 40 | 25 | 76 | 149 | 4688 | 88 | 67 |

**Table S2.** The space dynamics of the H9N2 HA protein in China from 1994 to 2019.

| Province | Total Number | Clade | | | | | | |
|---|---|---|---|---|---|---|---|---|
| | | **h9.4.2.1** | **h9.4.2.2** | **h9.4.2.3** | **h9.4.2.4** | **h9.4.2.5** | **h9.4.2.6** | **Unknown** |
| Jiangxi | 797 | 0 | 0 | 3 | 13 | 780 | 0 | 1 |
| Jiangsu | 603 | 19 | 7 | 6 | 2 | 563 | 0 | 6 |
| Guizhou | 520 | 0 | 0 | 0 | 0 | 515 | 5 | 0 |
| Guangdong | 505 | 4 | 3 | 5 | 11 | 523 | 62 | 3 |
| Shandong | 396 | 3 | 5 | 9 | 38 | 336 | 0 | 5 |
| Shanghai | 378 | 5 | 0 | 13 | 9 | 347 | 2 | 2 |
| Zhejiang | 237 | 1 | 0 | 0 | 0 | 236 | 0 | 0 |
| Anhui | 231 | 1 | 0 | 0 | 3 | 218 | 4 | 5 |
| Yunnan | 225 | 0 | 0 | 3 | 10 | 210 | 2 | 0 |
| Hubei | 195 | 1 | 0 | 0 | 2 | 191 | 1 | 0 |
| Ningxia | 181 | 0 | 0 | 0 | 1 | 180 | 0 | 0 |
| Fujian | 171 | 2 | 2 | 0 | 3 | 162 | 2 | 0 |
| Guangxi | 166 | 0 | 0 | 25 | 8 | 122 | 5 | 6 |
| Qinghai | 110 | 0 | 0 | 0 | 0 | 110 | 0 | 0 |
| Hunan | 94 | 0 | 0 | 0 | 1 | 69 | 1 | 23 |
| Xizang | 85 | 0 | 0 | 0 | 0 | 85 | 0 | 0 |
| Henan | 68 | 1 | 0 | 10 | 15 | 40 | 0 | 2 |
| Beijing | 40 | 0 | 2 | 0 | 3 | 30 | 0 | 5 |
| Hebei | 37 | 2 | 1 | 0 | 12 | 18 | 0 | 4 |
| Sichuan | 23 | 0 | 0 | 0 | 2 | 20 | 0 | 1 |
| Jilin | 13 | 0 | 0 | 0 | 3 | 9 | 1 | 0 |
| Liaoning | 13 | 0 | 0 | 0 | 4 | 7 | 2 | 0 |
| Tianjin | 11 | 0 | 2 | 0 | 0 | 9 | 0 | 0 |
| Heilongjiang | 9 | 1 | 1 | 0 | 2 | 3 | 0 | 2 |
| Shanxi | 8 | 0 | 0 | 0 | 0 | 8 | 0 | 0 |
| Xinjiang | 6 | 0 | 0 | 0 | 0 | 6 | 0 | 0 |
| Chongqing | 4 | 0 | 0 | 0 | 0 | 4 | 0 | 0 |
| Shaanxi | 3 | 0 | 0 | 0 | 0 | 3 | 0 | 0 |
| Hainan | 3 | 0 | 0 | 0 | 0 | 2 | 1 | 0 |
| Gansu | 3 | 1 | 0 | 0 | 1 | 1 | 0 | 0 |
| Taiwan | 3 | 0 | 0 | 0 | 0 | 3 | 0 | 0 |
| Inner Mongolia | 0 | 0 | 0 | 0 | 0 | 0 | 0 | 0 |

**Table S3.** The different amino acids of HA sequences around 2006.

| Group | Residue (H9 numbering) | | | | | | | | | | | |
|---|---|---|---|---|---|---|---|---|---|---|---|---|
| | **72** | **109** | **135** | **146** | **149** | **162** | **182** | **202** | **216** | **217** | **236** | **238** |
| **1994-2006 (230)** | E G<br>0.9%<br>98.7% | R S<br>1.3%<br>96.9% | G D<br>0.4%<br>97.4% | R Q<br>0%<br>95.6% | G N<br>0.4%<br>98.7% | Q R<br>3.1%<br>93.0% | R T<br>0%<br>97.8% | I T<br>0%<br>99.1% | L Q<br>44.3%<br>53.9% | M Q<br>0% 99.1% | K R<br>0.9%<br>99.1% | D N<br>7.9%<br>91.7% |
| **2007-2019 (4910)** | E G<br>64.4%<br>35.2% | R S<br>86.2%<br>10.7% | G D<br>55.2%<br>39.0% | R Q<br>56.7<br>41.3% | G N<br>59.6%<br>37.2.% | Q R<br>92.4%<br>6.2% | R T<br>53.7%<br>38.7% | I T<br>73.4%<br>20.4% | L Q<br>97.0%<br>2.8% | M Q<br>85.8%<br>14.0% | K R<br>81.9%<br>17.6% | D N<br>77.8%<br>21.9% |
| **GX/55** | G | S | D | Q | N | R | T | T | L | Q | R | N |
| **18325** | E | R | G | R | G | Q | R | I | L | M | K | D |
| **19027** | E | R | S | R | G | Q | R | I | L | M | K | D |
| **19166** | E | R | G | R | G | Q | R | I | L | M | K | D |

**Table S4.** Results of cross hemagglutination inhibition test.

| Strains | Antiserum | | | | | | | | |
|---|---|---|---|---|---|---|---|---|---|
| | **Mut-Mut/PR8** | **R-WT/PR8** | **WJ57** | **18325** | **HN98** | **F98** | **SD96** | **19027** | **19166** |
| **R-Mut/PR8** | 2048 | 256 | 128 | 128 | 256 | 512 | 32 | 256 | 512 |
| **R-WT/PR8** | 32 | 1024 | 1024 | 8 | 1024 | 2048 | 256 | 8 | 8 |
| **WJ57** | 128 | 2048 | 2048 | 64 | 1024 | 2048 | 256 | 64 | 64 |
| **18325** | 512 | 256 | 512 | 4096 | 256 | 512 | 128 | 2048 | 2048 |
| **HN98** | 4 | 256 | 512 | 4 | 256 | 256 | 32 | 4 | 4 |
| **F98** | 128 | 1024 | 2048 | 64 | 1024 | 4096 | 256 | 32 | 64 |
| **SD96** | 16 | 256 | 512 | 8 | 256 | 512 | 256 | 8 | 8 |
| **19027** | 256 | 128 | 64 | 1024 | 256 | 512 | 128 | 4096 | 1024 |
| **19166** | 256 | 32 | 64 | 256 | 128 | 128 | 64 | 1024 | 4096 |
